# The TRIOBP Isoforms and Their Distinct Roles in Actin Stabilization, Deafness, Mental Illness, and Cancer

**DOI:** 10.3390/molecules25214967

**Published:** 2020-10-27

**Authors:** Beti Zaharija, Bobana Samardžija, Nicholas J. Bradshaw

**Affiliations:** Department of Biotechnology, University of Rijeka, 51000 Rijeka, Croatia; beti.zaharija@biotech.uniri.hr (B.Z.); bobana.samardzija@biotech.uniri.hr (B.S.)

**Keywords:** TRIOBP, cancer, deafness, hearing loss, mental illness, schizophrenia, actin, cytoskeleton, disordered structure, protein aggregation

## Abstract

The *TRIOBP* (*TRIO* and *F-actin Binding Protein*) gene encodes multiple proteins, which together play crucial roles in modulating the assembly of the actin cytoskeleton. Splicing of the *TRIOBP* gene is complex, with the two most studied TRIOBP protein isoforms sharing no overlapping amino acid sequence with each other. TRIOBP-1 (also known as TARA or TAP68) is a mainly structured protein that is ubiquitously expressed and binds to F-actin, preventing its depolymerization. It has been shown to be important for many processes including in the cell cycle, adhesion junctions, and neuronal differentiation. TRIOBP-1 has been implicated in schizophrenia through the formation of protein aggregates in the brain. In contrast, TRIOBP-4 is an entirely disordered protein with a highly specialized expression pattern. It is known to be crucial for the bundling of actin in the stereocilia of the inner ear, with mutations in it causing severe or profound hearing loss. Both of these isoforms are implicated in cancer. Additional longer isoforms of TRIOBP exist, which overlap with both TRIOBP-1 and 4. These appear to participate in the functions of both shorter isoforms, while also possessing unique functions in the inner ear. In this review, the structures and functions of all of these isoforms are discussed, with a view to understanding how they operate, both alone and in combination, to modulate actin and their consequences for human illness.

## 1. Introduction

**Figure 1:**
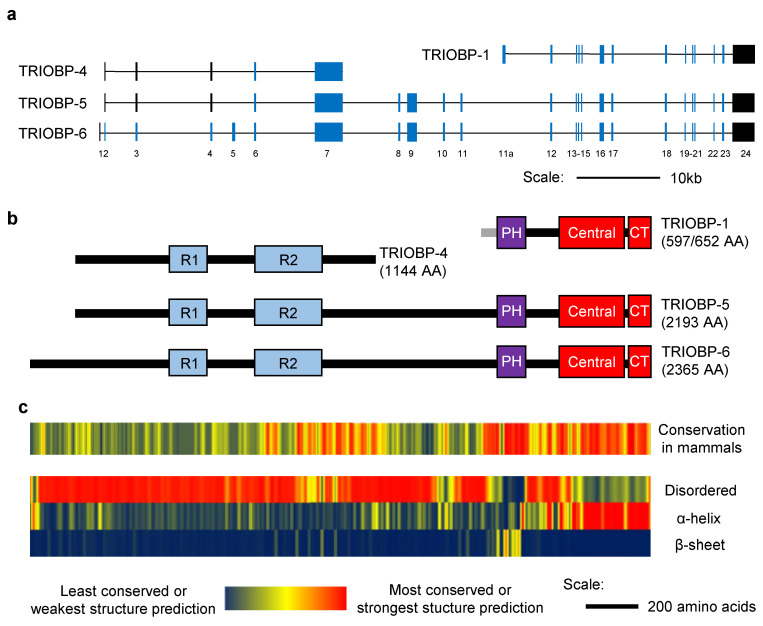
(**a**) Scale schematic of the alternative splicing of *TRIOBP* in humans. Exons (vertical bars) on the four most studied isoforms are shown, with introns represented by horizontal lines. Blue exons are entirely or mainly coding, black exons are entirely or mainly non-coding. Exon numbering is according to Park et al. [6]. (**b**) Scale schematic of the human TRIOBP-1, 4, 5, and 6 proteins with structural regions highlighted: R1, R2: First and second repeat domains, PH: Pleckstrin homology domain, Central: Central coiled coil domain, CT: C-terminal coiled coil domain. The number of amino acids (AA) in humans is also indicated. (**c**) The level of conservation of each section of the TRIOBP-6 amongst mammalian orthologues and predictions of three forms of secondary structure: disordered/unstructured protein, α-helix, and β-sheet. These are displayed as heat maps to scale with the schematic in part (**b**). Conservation determined using AL2CO [7], based on amino acid sequences of TRIOBP-6 (or similar splice variants) from 57 different mammalian genera. These were identified using BLAST (reference sequence human: TRIOBP-6, NP_001034230.1), aligned with CLUSTAL Omega 1.2.4 [8] and the alignment was then manually curated. Secondary structure predictions were made using PSIPDRED 4.0 and DISOPRED3 [9,10,11] with protein analyzed in three overlapping sections. All results were averaged over an 11 amino acid sliding window for clarity. The N-terminal 61 amino acids of TRIOBP-1 from exon 11a that are not present in TRIOBP-6 were not evaluated here, but were previously predicted to be disordered with comparatively poor conservation [12].

Actin filaments are one of the key elements of the cytoskeleton, and are vital for processes including cellular motility, neuronal differentiation, and cell–cell junctions. The core of these are composed of filamentous F-actin. These are formed by the polymerization of globular units of G-actin, and fibers can in turn depolymerize back to G-actin again [1]. The correct regulation of this key molecular process therefore impacts upon a wide array of cellular functions, and incorrect regulation is associated with various diseases [2]. Among the regulators of actin discovered in the last few decades are the proteins encoded by the *TRIO* and *F-actin Binding Protein* (*TRIOBP*) locus [3]. The *TRIOBP* gene is subject to complicated alternative splicing (Figure 1a). Multiple long splice variants exist [4,5], of which the longest is *TRIOBP-6*, although the slightly shorter *TRIOBP-5* is more often studied. The majority of published work into TRIOBP proteins, however, has instead focused on the products of two shorter transcripts. Of these, *TRIOBP-1* is transcribed from the 3′ end of the *TRIOBP* gene and encodes a largely structured protein [3] with a ubiquitous expression pattern [4,5]. In contrast, *TRIOBP-4* is transcribed from the 5′ end of the gene and encodes a structurally disordered protein, expressed predominantly in the inner ear and retina [4]. The TRIOBP-1 and TRIOBP-4 proteins share no common amino acid sequence, however, both share most or all of their primary structure with the longer variants (Figure 1b).

## 2. TRIOBP-1: A Structured Protein Implicated in Mental Illness and Cancer

### 2.1. The Structure of TRIOBP-1

TRIOBP-1 consists of two major structured regions: a predicted Pleckstrin homology (PH) domain near the N-terminus and coiled coil domains that make up the C-terminal half of the protein (Figure 1b). These are separated by a linker region of approximately 100 amino acids, referred to as the “mid domain” [13], which is predicted to be intrinsically disordered. Finally, TRIOBP-1 has an optionally translated disordered region at its extreme N-terminus, which is targeted to the nucleus of the cell [12]. This results from the existence of two different potential start codons, 59 amino acids apart from each other, and means that full length TRIOBP-1 can be either 593 or 652 amino acids in length [12]. The 593 amino acid long version of TRIOBP-1 was the first TRIOBP protein to be described, under the name TARA, for TRIO Associated Repeat on Actin [3] (also referred to as TAP68 [14]). This appears to be the more abundant species in many cell culture systems. The 652 amino acid long version may, however, be the principle TRIOBP-1 species in the human heart [15].

**Figure 2:**
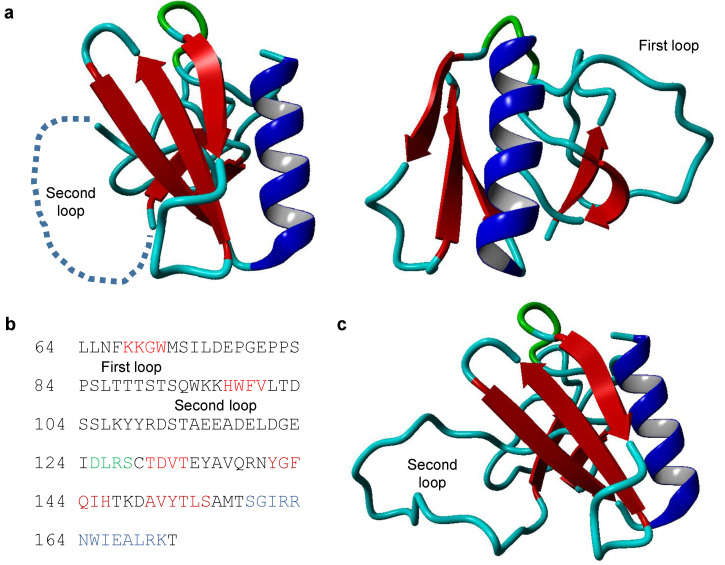
A structural homology model of the PH domain of TRIOBP-1 (amino acids 64–172 of 652 amino acid TRIOBP-1). (**a**) Images of the model, with the first loop region displayed. (**b**) Amino acid sequence of the PH domain, with the first and second loop regions indicated. Coloring corresponds to the secondary structures seen in the molecular images. (**c**) Image of the model including a low quality prediction of the strength of the second loop region. Model generated using MODELLER 9.20 [16], based principally on the structure of the PH domain of DAPP1 (PDB ID: 1FAO), which includes sequence analogous to the first loop region. Shorter sections including the second loop were modeled with additional templates (PDB ID: 2DYN, 2D9Y, 3GOC, and 5YUG). Alignments were generated using CLUSTAL Omega 1.2.4 [8], and then optimized manually. Of the 20 models generated, the one with the lowest objective function score was visualized using YASARA 18.4.24 [17].

The presence of a PH domain near the N-terminus is strongly predicted [3], and it was confirmed that this region of TRIOBP-1 forms a compact folded domain [12]. Its structure has never been studied experimentally, but based on homology with other proteins, it seems to be a fairly typical PH domain with two extended unstructured loops sticking out of it (Figure 2). These loops consist predominantly of polar and charged amino acids. The second, and larger, of these loops is highly conserved in mammals (Figure 1c). The function of the PH domain is currently unknown, however, it likely acts as a protein–protein interaction domain. No interaction of TRIOBP-1 with phosphoinositides has been published, although this cannot be formally discounted.

**Figure 3:**
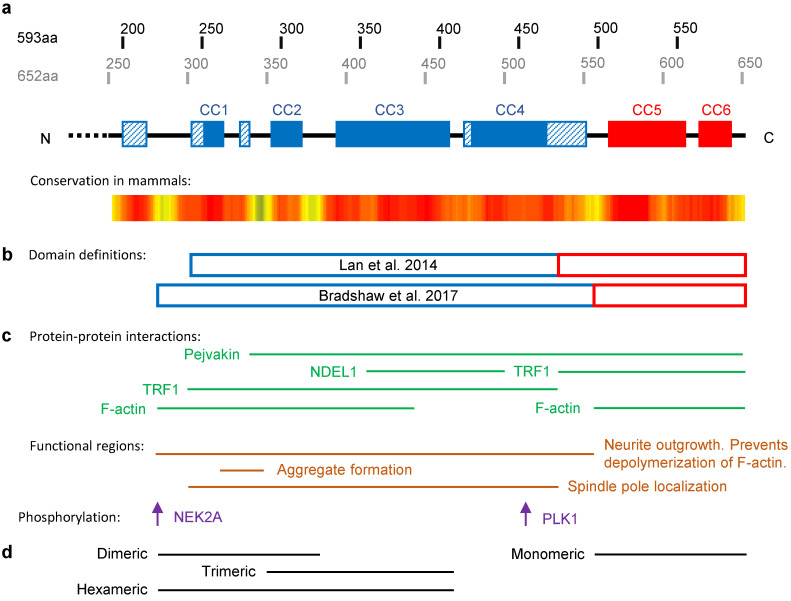
The structure of the coiled-coil regions of TRIOBP-1. All parts of this figure are to scale with each other. (**a**) Locations of predicted coiled-coils (CC). Solid filled boxes represent high confidence predictions, striped boxes represent lower confidence predictions, derived from PSIRPED [9]. CCs are colored based on their predicted inclusion in the central CC domain (blue) or C-terminal CC domain (red). Amino acid numbering from both the 593 amino acid and 652 amino acid TRIOBP-1 proteins are shown. Labeling of CCs is based on Bradshaw et al. [12] and differs from the numbering used by Katsuno et al. [18], who do not count the putative coiled-coil labeled here as CC1 in their numbering. Level of amino acid conservation is displayed using the same calculation and heat map as in Figure 1c. (**b**) Locations of constructs representing the central and C-terminal CC domains from two publications [12,14]. (**c**) Locations of regions of TRIOBP-1 involved in protein–protein interactions and functions [3,12,14,19,20]. Note that some proteins bind more than one region of TRIOBP-1. The only proteins so far reported to bind to TRIOBP-1 outside of these CC regions is TRIO, which binds to the mid domain between the central CC region and the PH domain [13]. The locations of two known phosphorylated residues and their associated kinases are also shown [14,21]. (**d**) Locations of fragments of TRIOBP-1 and the oligomeric states they adopt when expressed in isolation in vitro [12].

The C-terminal half of TRIOBP-1 is highly structured, having long been predicted to consist of coiled-coil (CC) domains (Figure 3a) [3]. Recent predictions suggest there to be approximately six CCs within this section of TRIOBP-1, which separate into two distinct domains: a larger central CC domain and a smaller C-terminal CC domain (Figure 3b) [12,14]. These two domains, and the central CC domain in particular, appear to be involved in many of the functions of TRIOBP-1 in the cell (Figure 3c). While the C-terminal CC domain is monomeric when expressed in isolation, the central CC domain forms an elongated hexamer, seemingly through distinct homodimeric and homotrimeric interactions (Figure 3d) [12]. The central CC domain is therefore responsible for the known oligomerization [22] of the full length TRIOBP-1 protein.

In addition to the main TRIOBP-1 species, which are approximately 70 kDa, smaller species have also been detected by western blotting, ranging in size from 45–60 kDa [15,23]. Based on the specificity of the antibodies used, these would be expected to represent the C-terminal 400–540 amino acids of TRIOBP-1, that is, the coiled-coil domains and variable amounts of the unstructured linker region, but not a complete PH domain. An additional splice variant, *TRIOBP-2*, has also been sequenced (annotated in genome assembly hg38), which encode the N-terminal sections of TRIOBP-1 including the PH domain and parts of the central coiled-coil domain. However, to date, this has not been thoroughly characterized.

### 2.2. TRIOBP-1 as a Regulator of Actin Polymerization

Upon its initial discovery, TRIOBP-1 was noted to adopt a filamentous expression pattern, appearing at 350 nm periodic intervals along the length of actin filaments [3]. Direct interaction between TRIOBP-1 and actin could be demonstrated in vitro, strongly indicating TRIOBP-1 to be an actin-associated protein [3]. Furthermore, TRIOBP-1 co-localizes with, although seemingly does not bind to, two other actin-associated proteins, actinin and myosin II [3]. Knockdown of TRIOBP-1 by siRNA has repeatedly been shown to lower the expression of filamentous F-actin in cell systems [24,25,26], while over-expression of TRIOBP-1 in cell lines leads to a “cell spreading” phenotype, resulting from excessive F-actin formation [3]. Notably, the central CC domain of TRIOBP-1 is capable of interacting with F-actin and blocking its depolymerization into G-actin [12]. One of the principle cellular functions of TRIOBP-1 therefore appears to be maintaining the existence of F-actin fibers.

In wound healing assays, performed in neuroblastoma cells, overexpression of TRIOBP-1 was seen to increase the rate of cellular migration [19]. This effect was cumulative with that of overexpressing NDEL1 (Nuclear Distribution Element-Like 1, also known as Nudel) [19]. NDEL1 is a key neurodevelopmental protein with links to mental illness, which is more commonly associated with the microtubule cytoskeleton [27]. Nevertheless, NDEL1 directly interacted with TRIOBP-1, binding to the central CC region at approximately the fourth coiled coil, and appeared to work co-operatively with TRIOBP-1 to enhance levels of F-actin [19]. Furthermore, in neuronal systems, TRIOBP-1 appears to recruit two key kinases to NDEL1 [28]. The ensuing phosphorylation events lead to increased F-actin formation, neurite outgrowth, and dendritic arborization [28]. TRIOBP-1 and NDEL1 therefore appear to act synergistically in cell migration and neuronal differentiation. 

Another important role of actin is in relation to the receptors that modulate adhesion between the cell and both its extracellular matrix and other cells. The actin cytoskeleton physically links these and provides the basis of mechanical force within the cell that allows it to interact with external stimuli [29]. TRIOBP-1 has been identified in the focal adhesions that link cells to the extracellular matrix, and its expression there is regulated by myosin II [30], which generates tension, leading to maturation of the focal adhesions. TRIOBP-1 is also found at the adhesion junctions between cells [13]. In adhesion junctions of epithelial cells, expression of the crucial transmembrane protein E-cadherin is regulated by the RhoGEF TRIO. TRIOBP-1 binds to TRIO using its mid domain and prevents this effect, leading to increased E-cadherin expression and increased density of actin filaments [13]. It remains to be clarified whether this role of TRIOBP-1 in modulating actin via TRIO is distinct from its effect on actin depolymerization, which seems to occur through direct binding [3,12].

TRIOBP-1 is also found at the adherens junctions in the heart, where it interacts with JCAD (Junctional Protein Associated with Coronary Artery Disease) [31]. Knockdown of either TRIOBP-1 or JCAD in epithelial cells led to reduced F-actin stress fiber formation [31]. TRIOBP-1 also possesses an additional function in the heart through its interaction with the voltage gated ion channel hERG1 (human Ether-à-go-go-Related Gene 1, also known as KCNH2) [15]. In cardiomyocytes, TRIOBP-1 affects expression of hERG, with direct effects on cardiac rapidity, leading the authors to speculate that TRIOBP-1 may function as a bridge between actin filaments and hERG1 in the membrane, linking excitation of the ion channel to cell mobility [15]. 

### 2.3. TRIOBP-1 in the Cell Cycle

TRIOBP-1 is essential for correct mitotic progression, with its knockdown in cells leading to multipolar spindle formation [14]. Similar effects are also observed when expression levels of TRIOBP-1 expression were increased, through knockdown of ubiquitin ligase HECTD3 [32]. This suggests that regulation of TRIOBP-1 expression is of significant importance. The most likely mechanism by which TRIOBP-1 affects mitotic progression is through its interaction with TRF1 (Telomere Repeat Factor 1 [22,33]). TRF1 is found at the telomeres of cells, and is involved in both telomere stability and cell cycle regulation. Notably, the localization of TRF1 during mitosis is dependent on that of TRIOBP-1 [14]. The localization of TRIOBP-1 during the cell cycle is itself regulated by two kinases, with PLK1 in particular being required for both its localization in prophase and metaphase, and also for its interaction with TRF1 [14,21]. Strikingly, mutation of the threonine in TRIOBP-1 that is phosphorylated by PLK1 leads to mitotic arrest in prometaphase [21]. Specifically, the chromosomes fail to segregate, highlighting the importance of TRIOBP-1 in this process. While there is some evidence that actin plays a role in mitosis, it remains to be determined whether the function of TRIOBP-1 in mitosis is directly related to its F-actin stabilization effect.

### 2.4. TRIOBP-1 in Mental Illness

It has recently been suggested that chronic mental illnesses such as schizophrenia, bipolar disorder, and major depression may be caused in part by the accumulation of aggregates of specific proteins in the brains of patients [34,35], in partial analogy to similar insoluble protein deposits in neurodegenerative conditions. In order to detect such proteins, the total insoluble (and aggregated) protein fraction was isolated from the brains of patients with schizophrenia, and used to inoculate a mouse. Monoclonal antibodies were generated from this animal and screened for the ability to specifically recognize the insoluble protein fraction of the patient brain compared to an equivalent preparation from the control brain tissue [36]. One such antibody was found to recognize TRIOBP-1, suggesting it to be present in an aggregated state in the brains of at least a subgroup of patients [23].

TRIOBP-1, but not TRIOBP-4, formed insoluble aggregates when expressed in mammalian cell culture or rodent primary neurons [23]. Subsequent mapping studies determined the central CC region of TRIOBP-1 to be the basis of its aggregation propensity [12]. The critical region for aggregation has now been mapped to a 25 amino acid long loop containing multiple charged amino acids [12]. In addition to 70 kDa full length TRIOBP-1, aggregation is also seen of shorter (45–60 kDa) protein species, representing coiled-coil regions of TRIOBP-1, but without the PH domain [23]. The consequences of TRIOBP-1 aggregation are still being determined, although effects have been seen on neurite outgrowth in cell culture [23]. Structures resembling aggregates have also been seen when TRIOBP-1 is expressed in other tissues [15,20]. Regulation of *TRIOBP* expression and folding may therefore be important for mental health. One such regulatory factor is already known, the ubiquitin ligase HECTD3, which leads to degradation of TRIOBP-1 [32].

Unlike several other proteins that are implicated as aggregating in mental illness [35], TRIOBP-1 is not encoded for by a known genetic risk factor for major mental illness. This may be because the functions of TRIOBP-1 in actin regulation are fundamental to life, and as such, mutations in its (highly conserved) sequence would lead to outcomes more detrimental than those seen in mental illness. Supporting evidence comes from a handful of studies, however. First, in two screens of samples from separate brain banks, levels of *TRIOBP* transcripts were seen to be subtly, but significantly higher in schizophrenia patients than in the controls [37]. Second, a polymorphism in the *NDE1/miR-484* locus, previously associated with schizophrenia in the Finnish population [38], was found to affect the expression of *TRIOBP* transcripts [39,40]. *MiR-484* expression was subsequently shown to lead to increased levels of the TRIOBP-1 protein [40]. Finally, a consanguineous family has been reported who suffer from schizophrenia, epilepsy, and hearing, with linkage to chromosome 22q12.3 q13.3 [41]. It is therefore possible, although not yet verified, that rare variants in *TRIOBP* could be responsible for these phenotypes.

### 2.5. TRIOBP-1 in Cancer

TRIOBP-1 has been identified in cell lines from a range of different cancers including lung carcinoma [42], glioblastoma [43], esophageal [44], pancreatic [45], prostate, lung, and breast cancer [46]. Studies with glioblastoma showed that TRIOBP (from the specificity of the antibody used: TRIOBP-1, 5, and/or 6) was more abundant in the tumors themselves than in the surrounding tissues [43]. Analysis of existing datasets suggested that it was also over-expressed in classical, mesenchymal, neuronal, and pro-neuronal glioblastoma [43]. Further analysis in glioblastoma cell lines demonstrated that knockdown of TRIOBP-1 (and TRIOBP-5/6) reduced the proliferation and migration of these cells [43].

Another interesting line of research comes from study of the microRNA *miR-3178*, a target of the cancer-suppressing protein SP1, which was shown to have anti-metastatic properties in a mouse model [46]. *MiR-3178* inhibits the expression of *TRIOBP-1* and *5*, as measured at both the transcript and protein levels, through binding to their untranslated 3’ exon. Crucially, while *miR-3178* inhibits the migration and integration of metastatic cells, this effect can be reversed by expression of TRIOBP-1 [46]. Together, there is therefore evidence that TRIOBP-1 affects tumor metastasis through its known roles in actin modulation as well as potentially through its roles in the cell cycle.

### 2.6. TRIOBP-1 in Other Diseases

While TRIOBP-1 is not generally considered to have a significant role in hearing loss, unlike TRIOBP-4, it should be noted that TRIOBP-1 is expressed in the stereocilia of the inner ear [20]. Here, it binds to the hearing-related protein Pejvakin, with over-expression of TRIOBP-1 causing Pejvakin to form aggregates [20]. There have also been reported missense mutations within *TRIOBP-1* in patients with hearing loss [47,48] (Table 1), however, these would also affect longer splice variants of *TRIOBP*.

## 3. TRIOBP-4: A Disordered Protein Implicated in Deafness

### 3.1. The Structure of TRIOBP-4 

**Figure 4:**
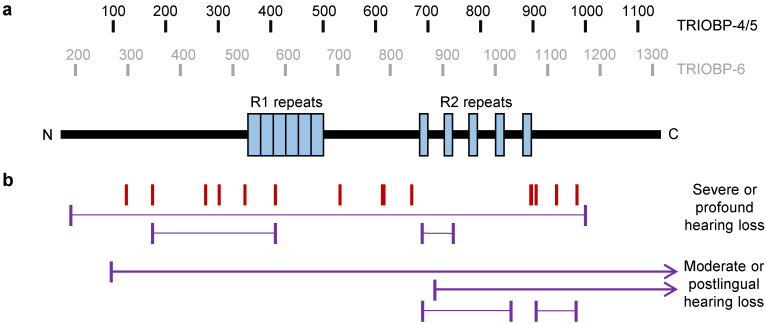
(**a**) The location of the repeats that make up the R1 and R2 regions of TRIOBP-4, with ¸amino acid numbering of both TRIOBP-4/5 and TRIOBP-6. (**b**) The location of frameshift and nonsense mutations from patients with hearing loss. Red bars indicate homozygous mutations, while purple bars joined by dotted lines indicate compound heterozygous mutations. Arrowheads indicate that the other heterozygous mutations lie in a region of *TRIOBP-5/6* that is 3′ of the *TRIOBP-4* open reading frame. Full details of these are in Table 1. All elements of this figure are shown to scale.

Human TRIOBP-4 is a 1144 amino acid long protein, which is predicted to be almost entirely disordered, possessing no fixed secondary or tertiary structure [49]. While TRIOBP-4 therefore possesses no folded domains, it has been observed to contain two repeat regions [4], referred to as R1 and R2 (Figure 4a) [49]. The R1 repeat region lies near the center of the protein. In humans, it has a high isoelectic point of 11.7 and consists of six repeats (with slight variations) of the sequence SSPNRTTQRDNPRTPCAQRDNPRA [49]. R2, in humans, consists of five repeats of the sequence VCIGHRDAPRASSPPR (with slight variations), with 30–40 amino acids between each repeat. It lies in the C-terminal half of TRIOBP-4 and has a much lower isoelectric point of 5.4 [49].

### 3.2. TRIOBP-4 as an Actin Bundling Protein in the Inner Ear

TRIOBP-4 binds directly to F-actin, principally through its R1 repeat domain, and is found along the length of filaments [49,50]. R2 shows a considerably weaker, probably hydrophobic interaction to actin [49]. In vitro assays showed TRIOBP-4 molecules to bind actin subunits at a ratio of 1:3–1:4, and that addition of TRIOBP-4 caused actin filaments to become organized into densely packed bundles, which resembled the hair cell rootlets of the inner ear [50].

TRIOBP-4 has a very specialized expression pattern and is highly expressed in the hair cells of the inner ear [50]. These cells perform mechano-electrical transduction from the fluid motion that is induced by sound into neuronal signaling. This occurs through stereocilia, organelles containing an F-actin core, which are anchored into the cuticular plate of hair cells by rootlets, and which pivot in response to fluid motion. TRIOBP-4 is found in the upper sections of these rootlets as well as along the length of the stereocilia themselves in their actin cores [18,50]. TRIOBP-4 is also found in Deiters’ cells, which support the hair cells [18]. Normally, stereocilia rootlets would form in the first 16 postnatal days of mice, however, they were not seen to form at all in mice lacking the ability to produce either TRIOBP-4 or the longer isoforms (homozygous deletion of mouse exon 6, equivalent to human exon 7, Figure 1a) [50]. While stereocilia still form, they are considerably less rigid than those of wild type animals, often being found pointing in the wrong direction, and progressively degenerate [50]. These stereocilia still react to mechano-electrical transduction, but no longer have the rigidity required to remain upright and pivot in response to sound [50]. Seemingly as a result of this, these mice are profoundly deaf [50]. It therefore appears that the actin bundling function of TRIOBP-4 is crucial for the formation of the stereocilia rootlet and forming them into tight actin bundles, which are required for their stability and rigidity [18,50].

While the known role of TRIOBP-4 as an acting bundling protein has been largely restricted, so far, to studies in the inner ear, a more general role for it is suggested by two lines of evidence. First, while *TRIOBP-4* does show a very specialized expression pattern, it is not unique to the inner ear, with its transcripts notably being highly expressed in the retina [4]. Second, knockdown of TRIOBP-4 in a pancreatic cancer cell line led to reduced filopodia formation, with TRIOBP-4 seen at actin bundles of these structures [45].

### 3.3. TRIOBP-4 in Hearing Loss

In 2000, details were reported of a Palestinian family with nonsyndromic hereditary deafness, linked to a locus on chromosome 22, which was labeled as DFNB28 [5]. Homozygosity mapping implicated the *TRIOBP* locus, but no mutations were found in *TRIOBP-1*, the only open reading frame of *TRIOBP* known at that time. This directly led to the cloning of the long splice form *TRIOBP-5* and the discovery of a homozygous nonsense mutation within it [5] as well as separate mutations in other families with nonsyndomic deafness [5]. Simultaneously, studies of deafness linked-loci in families from India and Pakistan led to the discovery of a range of other *TRIOBP* mutations as well as cloning of *TRIOBP-4* and *6* [4]. Subsequently, a large number of studies have sequenced the *TRIOBP* gene in families or individuals with severe or profound prelingual hearing loss, revealing a wide range of seemingly pathogenic recessive mutations (Table 1, Figure 4b). These pathogenic mutations tend to be homozygous in patients with deafness, in many instances as a result of consanguinity. Patients have also been found with compound heterozygous expression of two different *TRIOBP* mutations.

The majority of mutations detected to date in patients are either nonsense or frameshift mutations in *TRIOBP-4*, which would lead to the expression of truncated TRIOBP-4 and longer splice variants such as TRIOBP-5 and 6, but with no predicted effect on TRIOBP-1. While many of the mutations lie with the large exon 7 (as in *TRIOBP-6*, Figure 1a), their location in the TRIOBP-4 protein varies considerably (Figure 4b). Many, but not all, of the predicted truncated proteins would still contain the R1 repeat region. Most either lack the R2 region, or would only partially express it. It is likely that these putative truncated proteins would be non-functional and degraded by the proteasome. Deafness in patients with these mutations therefore likely arises through lack of functional TRIOBP-4, which is consistent with the finding that mice lacking TRIOBP-4 (and longer isoforms) are profoundly deaf [50]. An alternative hypothesis would be that loss of the R2 region and/or C-terminal region of TRIOBP-4 would lead to expression of truncated proteins, which could interfere with normal stereocilia function. While the roles of these regions are not well characterized, it is notable that they are among the most highly conserved regions of *TRIOBP-4* in mammals (Figure 1c). In both instances, based on studies in mice [18], it is likely that stereocilia rootlets fail to form in the patients, leading to degeneration of stereocilia and thus hearing loss. Consistent with this, some patients with TRIOBP-4 mutations and hearing loss have had been successfully treated using cochlear implants, which bypass the need for stereocilia [50,51].

While most *TRIOBP* mutations implicated in deafness were found in patients with severe or profound hearing loss detected before speaking, compound heterozygous mutations have been reported in patients with moderate hearing loss or later onset severe hearing loss (Table 1). Notably, many of these patients either possess a mutation that lies 3′ of the *TRIOBP-4* reading frame, affecting longer splice variants only, or else have a mutation near the C-terminus of TRIOBP-4, meaning that the R2 domain would still be intact (Figure 4b). Potentially, the presence of some TRIOBP-4 functionality could therefore explain the milder phenotype, although there are instances of similar C-terminal mutations in patients with severe hearing loss. 

Genome wide association studies have also shown that intronic SNP rs58389158 is associated with age related hearing impairment in non-Hispanic white individuals from California [52]. This SNP lies in an intron common to *TRIOBP-4*, *5*, and *6*. It is close to, and correlates strongly with, the coding SNP rs5756795, which leads to an F1187I protein variant [52]. The rs58389158 finding was replicated in the UK Biobank [52], in which rs5756795 was also found to be associated with both hearing difficulty and hearing aid use at the genome-wide level [53]. Common sequence variants in *TRIOBP* therefore appear to have an impact on hearing, in addition to rare nonsense and frameshift mutations.

### 3.4. TRIOBP-4 in Cancer

In contrast to the general expression of *TRIOBP-1* and, to a lesser extent, longer *TRIOBP* splice variants in cancer [46], *TRIOBP-4* transcripts were specifically seen to be expressed in a cancer cell line, HPAC [45]. Subsequent analysis found TRIOBP-4 to be upregulated in human pancreatic and, to an extent, breast cancer tissue, but not in prostrate or lung cancer tissue. Knockdown of TRIOBP-4 (and longer variants) in several pancreatic cancer cells lines led to a reduction in cell proliferation [45]. Therefore, it appears that TRIOBP-4 may play a specialized role in pancreatic cancer.

Additionally, a T195I missense mutation in TRIOBP-4 was among several mutations detected in a family with seemingly genetic, gastric, and rectal cancer [62]. Subsequent exome sequencing of additional families with these diseases led to the identification of several additional missense mutations in patients, two of which, A660V and S826L, segregated with disease in families [62]. These would also effect longer *TRIOBP* splice forms, and it remains to be confirmed whether they are pathogenic.

### 3.5. TRIOBP-4 Mutations in Other Illnesses

In addition to hearing loss, rare missense mutations in *TRIOBP-4* (and longer splice variants) have also been detected in a patient with multiple sclerosis (A322S mutation) [63] and in a patient with developmental delay, visual impairment, muscle weakness, hypotonia, clinodactyly, and mild hearing impairment (R1078C mutation) [64].

## 4. Potential Significance of the Longer Splice Variants TRIOBP-5 and TRIOBP-6

### 4.1. The Structure of the Long Splice Variants

While TRIOBP-1 and 4 share no common amino acid sequence with each other, they do with the longer TRIOBP splice variants. These contain the entire coding sequence of TRIOBP-1 and 4, except for the optionally translated extreme N-terminus of TRIOBP-1 (Figure 1). In human, the longest isoform, TRIOBP-6, is derived from a 24 exon long transcript, of which all but exons 1 and 24 are coding. This leads to a 2365 amino acid peptide, which forms the basis of numbering for all *TRIOBP* putative pathological mutations (Table 1). The majority of biological experiments, however, have instead focused on TRIOBP-5 (also called TRIOBP-3 in some earlier articles), which is a 2193 amino acid protein in humans. TRIOBP-5 is also the longest established isoform in mice. It derives from a transcript lacking exons 1 and 5, and whose open reading frame only begins on exon 6. This is because the Kozak sequence used for *TRIOBP-6* is encoded across exons 1 and 2, and is therefore incomplete in *TRIOBP-5* transcripts. As a result, TRIOBP-5 begins its reading frame at the same point as TRIOBP-4, but is otherwise identical in the amino acid sequence to TRIOBP-6.

TRIOBP-6 has some isoform-specific amino acid sequence at its N-terminus, plus both it and TRIOBP-5 share some of the coding sequence, which lies between the coding exons of *TRIOBP-1* and *5* (Figure 1a,b). These additional sequences are predicted to be predominantly unstructured, with the exception of a possible short stretch of α-helix in the isoform specific N-terminus of TRIOBP-6, and another near the center of the long isoforms (Figure 1c). Neither of these regions show significant sequence similarity to known protein structures. The long variants are therefore predicted to be intrinsically disordered for most of their length, but with the PH domain and coiled-coil domains of TRIOBP-1 at their C-terminal ends. The coiled-coil regions of TRIOBP-5 have been shown to lead to oligomerization, in a similar manner to TRIOBP-1 [12,18]. These proteins also possess multiple actin binding domains, sharing both the R1 repeat of TRIOBP-4 and coiled-coil domains of TRIOBP-1.

### 4.2. TRIOBP-5 in the Inner Ear and Deafness

While the majority of *TRIOBP* mutations found in patients with profound hearing loss lie within the reading frame of *TRIOBP-4* (Table 1), these would also affect the TRIOBP-5 and 6 proteins. Additionally, patients with moderate and/or progressive hearing loss have been described that possess both a mutation in *TRIOBP-4* and a p.G1672* mutation on the other *TRIOBP* allele, which would affect only the longer splice variants [59,61]. Consistent with this, while mice who lack both TRIOBP-4 and 5 show profound deafness [50], those engineered to express TRIOBP-4, but not 5, instead display a progressive form of deafness [18]. Together, these findings strongly imply that while TRIOBP-4 is essential for prelingual hearing ability, specific loss of the longer splice variants is also required for maintenance of hearing.

TRIOBP-5 is expressed in the same inner ear cell types as TRIOBP-4, with both being found in the stereocilia rootlets [50]. In contrast to TRIOBP-4, however, TRIOBP-5 is predominantly found in the lower parts of the rootlet, below the apical surface [18]. The specific role of TRIOBP-5 in the ear has been studied using various *TRIOBP*-deficient mice. While deletion of both *TRIOBP-1* and *TRIOBP-5* is lethal [50], mice lacking two *TRIOBP-5* specific exons are viable, as are heterozygous mice that can express TRIOBP-1 from one allele and TRIOBP-4 from the other [18]. These *TRIOBP-5*-deficient mice still express TRIOBP-4 in the stereocilia and retain residual hearing for at least 4–8 weeks [18]. This contrasts with the profound deafness of *TRIOBP-4*-deficient mice [50], indicating a unique role of TRIOBP-5, which is also essential for hearing. Detailed analysis of the *TRIOBP-5* knockout mice revealed that stereocilia appear to form normally, but then become increasingly disorganized over time. Specifically, some fuse together or are missing, while others appear thin and fragmented compared to those of wild-type animals [18]. The stereocilia are also seen to be less stiff, and to rotate less freely than wild-type ones [18]. Therefore, while TRIOBP-4 appears to be required to form stereocilia rootlets and elongate them into tight actin bundles (a role indispensable for hearing), TRIOBP-5 instead plays a separate, later role in widening and giving structure to the stereocilia (loss of which leads to progressive hearing loss) [18,50]. 

Interestingly, this role of TRIOBP-5 in modeling of the rootlets is retained in mice that express incomplete TRIOBP-5 (terminating after the PH domain), however, they do not gain the usual resilience [18]. Such mice may therefore reflect patients with mutations like p.G1672*, who have moderate progressive hearing loss, but not the profound hearing loss associated with mutations in *TRIOBP-4* [18,59,61]. This also implies that the role of TRIOBP-5 in the stereocilia is likely to involve its coiled-coil domains. One possible explanation for this is that these domains interact with Pejkavin, a protein also required for bundling of actin in the inner ear and for hearing, which was seen to interact with this region of TRIOBP-1 [20,65].

It is likely that TRIOBP-6 could also be involved in this process, but this remains untested due to lack of a known murine TRIOBP-6 species.

### 4.3. Potential Significance for the Long Splice Variants in Other Processes and Diseases

TRIOBP-5 and/or 6 are known to be expressed in the brain alongside TRIOBP-1 [4,5], and TRIOBP-5 exogenously expressed in neurons forms aggregates similar to those of TRIOBP-1 [23]. It is therefore possible that aggregation of longer TRIOBP isoforms may play a role in mental illness, but this remains to be investigated.

*TRIOBP-5* and/or *6* was also seen to be upregulated in a pancreatic cancer cell line, distinct from another cell line that expressed *TRIOBP-4* in the same study [45]. Curiously, knockdown of *TRIOBP-5/6* in these cells led to reorganization of the actin cytoskeleton and inhibition of filopodia formation [45]. This implies the existence of a more general role for TRIOBP-5/6 in actin dynamics, of potential relevance for cancer. This may occur through its actin binding sites in either repeat region R1 shared with TRIOBP-4, its central coiled coil domain shared with TRIOBP-1, or a combination. One piece of evidence arguing for a TRIOBP-4-like mechanism is that, in a wound healing assay, knockdown of *TRIOBP-5/6* led to reduced cell motility, but this could be rescued through expression of TRIOBP-4 [45]. However, TRIOBP-5/6 was also seen, along with TRIOBP-1, to have its expression inhibited by the metastasis suppressing microRNA *miR-3178*, suggesting that a TRIOBP-1-like role of the longer isoforms also exists, and is of relevance to cancer [46].

## 5. Conclusions and Unanswered Questions

**Figure 5:**
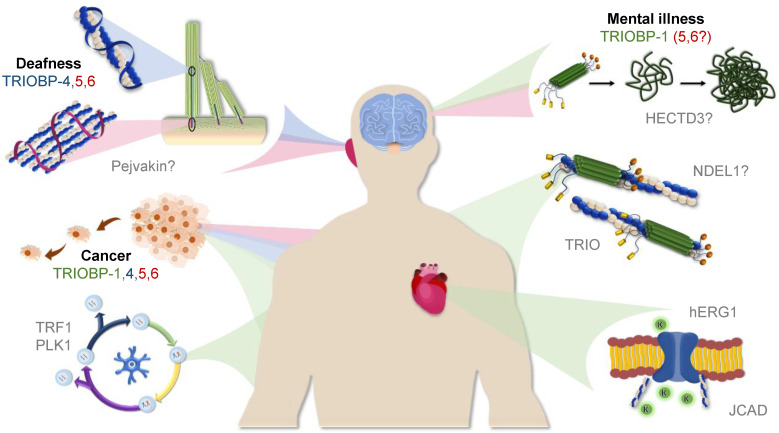
Illustrated representation of the expression and roles of TRIOBP-1 (green), TRIOBP-4 (blue), and TRIOBP-5/6 (red). Clockwise from top: Aggregation of TRIOBP-1 in the brain and mental illness; Role of TRIOBP-1 in F-actin stabilization throughout the body; Linking of actin to ion channel function in the heart by TRIOBP-1; role of TRIOBP-1 in the cell cycle; Importance of all major TRIOBP isoforms in metastasis; Distinct roles of TRIOBP-4 and TRIOBP-5 in the stereocilia of the inner ear and deafness. Protein interaction partners implicated in the various processes are indicated in gray.

The *TRIOBP* locus therefore encodes a variety of distinct proteins (Figure 1) with TRIOBP-1 being a structured and ubiquitously expressed protein implicated in mental illness and TRIOBP-4 being a disordered protein with specialized expression pattern essential for hearing. The long isoforms TRIOBP-5 and 6 combine the structures and many of the functions of the shorter isoforms, but with distinct additional roles in the ear, and potentially elsewhere. In spite of this, all the isoforms are linked through their role in stabilizing actin (Table 2, Figure 5).

The role of TRIOBP-1 in actin dynamics appears to be to bind directly to F-actin [3] and inhibit its depolymerization [12]. This appears to be a general function of TRIOBP-1 in many cell types and organs, with specific roles including the linking of adhesion receptors at the cell surface [13,30], neuronal outgrowth [28], cell migration [19], and signal transduction to mechanical force in the heart [15]. An additional, or possibly alternative, mechanism is that TRIOBP-1 can affect actin in certain circumstances through inhibition of TRIO [13]. TRIOBP-5 and/or 6, which share all the functional domains of TRIOBP-1, are also present in the brain and so can be presumed to participate in many functions there, but not in other TRIOBP-1 expressing tissues such as the heart or liver [4,5]. In contrast, TRIOBP-4 is an actin-bundling protein that likely uses its lack of rigid structure to wrap around actin fibers in the stereocilia, binding using its R1 repeat motif, and bundle them together during rootlet formation and early stereocilia development [45,50]. TRIOBP-5 (and possibly TRIOBP-6) then has a similar, but distinct role, in which it further “sculpts” and maintains the actin core of the stereocilia [18]. TRIOBP-1 is also present in the inner ear and stereocilia [4,5,20], but is seemingly not required for stereocilia formation or hearing [18].

An area where the *TRIOBP* isoforms show greater overlap is in the pathology of cancer. *TRIOBP-1* is expressed in many cancer cells and tissues, while *TRIOBP-4* and *5/6* are more specialized, in partial analogy to their normal expression patterns [42,43,44,45,46], although not necessarily in the same tissue types. Notably, all are implicated in metastasis. Specifically, suppression of TRIOBP-1 and 5/6 expression appears to be a means through which *miR-3178* suppresses metastasis [46], while TRIOBP-4 and 5/6 are implicated the in cell motility of pancreatic cancer cells [45].

While much has therefore been uncovered regarding these proteins, many questions remain. The exact mechanism through which TRIOBP-1 modulates actin is still only partially understood, and its roles in various organs and cell types need further analysis. While both its potential roles in mental illness and cancer are tantalizing, the relationship between it and specific mental illnesses and cancer subtypes needs to be established in larger patient samples. The role of TRIOBP-4 in the stereocilia is understood in more detail, and the role of frameshift and nonsense mutations of *TRIOBP-4* in hearing loss is well established. Nevertheless, the apparent role of more common variants of *TRIOBP-4* in hearing remains to be explored, as does its function in the retina. Perhaps the largest unexplored area of TRIOBP research, however, concerns the other splice variants. Little is known about shorter 3′ variants such as *TRIOBP-2*. For longer variants, putative roles have been found in the inner ear, but their role in the brain and other tissues is unclear, as is the relationship between TRIOBP-5 and TRIOBP-6.

The *TRIOBP* locus therefore provides a fascinating example of how multiple parts of a gene can cooperate in a single function, actin stabilization, through the generation of many different functional splice variants with distinct expression patterns and modes of action. The variety of different human diseases and conditions related to it highlights its importance, however, much work still needs to be done to clarify the exact relationships between these isoforms and with human health.

## Figures and Tables

**Table 1:** Published mutations in *TRIOBP* from individuals and families with hearing loss.

Mutation ^1^	Type ^2^	Zygosity ^3^	Isoforms (Location) ^4^	Origin ^5^ of Proband(s)	Ref (s)
Severe to Profound Hearing Loss					
p.P191Rfs*50	FS	CHT (p.P1172Cfs*13)	4, 5, 6	South Africa	[54]
p.Q297*	NON	HM	4, 5, 6	India	[4]
p.R347*	NON	HM CHT (p.Q581*)	4, 5, 6	Palestinian Palestinian	[5] [5]
p.R448*	NON	HM	4, 5, 6	China, Afghan	[51,55]
p.R474*	NON	HM ^7^	4, 5, 6	Pakistan ^7^	[50]
p.R523*	NON	HM ^7^	4, 5, 6	Pakistan ^7^	[50]
p.Q581*	NON	HM CHT (p.R347*) CHT (p.G1019R)	4, 5, 6 (R1)	Palestinian Palestinian Palestinian	[5] [5] [5]
p.Q740*	NON	HM ^7^	4, 5, 6	Pakistan ^7^	[50]
p.R785Sfs*50	FS	HM	4, 5, 6	Turkey	[56]
p.R788*	NON	HM	4, 5, 6	Pakistan	[4]
p.R841*	NON	HM	4, 5, 6	Turkey	[54]
p.R861*	NON	CHT (p.R920*)	4, 5, 6 (R2)	China	[57]
p.R920*	NON	CHT (p.R861*)	4, 5, 6 (R2)	China	[57]
p.G1019R	MIS	CHT (p.Q581*)	4, 5, 6 (R2)	Palestinian	[5]
p.I1065V	MIS	CHT (p.R1982H)	4, 5, 6 (R2)	China	[48]
p.R1068*	NON	HM	4, 5, 6 (R2)	Pakistan, Iran	[4,58]
p.D1069fs*12	FS	HM	4, 5, 6 (R2)	India	[4]
p.R1078Pfs*6	FS	HM	4, 5, 6 (R2)	India	[4]
p.R1117*	NON	HM	4, 5, 6	India	[4]
p.E1156*	NON	HM^7^	4, 5, 6	Pakistan ^7^	[50]
p.P1172Cfs*13	FS	CHT (p.R191Rfs*50)	4, 5, 6	South Africa	[54]
p.R1982H	MIS	CHT (p.I1065V)	1, 5, 6	China	[48]
p.S2121L	MIS	HM	1, 5, 6 (Centr.)	Iran	[47]
Moderate or Postlingual Hearing Loss ^6^					
p.Q268Lfs*432	FS	CHT (p.G1672*)	4, 5, 6	Poland	[59]
p.R861*	NON	CHT(p.P1030Lfs*183)	4, 5, 6 (R2)	USA, Iran	[54,60]
pR885Afs*120	FS	CHT (p.G1672*)	4, 5, 6	Netherlands	[61]
p.P1030Lfs*183	FS	CHT (p.R861*)	4, 5, 6 (R2)	USA, Iran	[54,60]
p.R1078Pfs*6	FS	CHT (p.L1154Afs*29)	4, 5, 6 (R2)	Netherlands	[61]
p.M1151V	MIS	CHT (p.P1396R)	4, 5, 6	China	[57]
p.L1154Afs*29	FS	CHT (R1078Pfs*6)	4, 5, 6	Netherlands	[61]
p.P1396R	MIS	CHT (p.M1151V)	5, 6	China	[57]
p.G1672*	NON	CHT (p.Q268Lfs*432) CHT (pR885Afs*120)	5, 6	Poland Netherlands	[59] [61]

^1^ Amino acid number of human TRIOBP-6, NM_001039141.2 (for amino acid locations in TRIOBP-4 or 5, subtract 172 amino acids). ^2^ FS: Frameshift, NON: Nonsense, MIS: Missense. ^3^ HM: Homozygous, HT: Heterozygous, CHT: Compound heterozygous with the mutation indicated. ^4^ Numbers refer to isoforms, e.g., “5,6” indicates the mutation lies within TRIOBP-5 and TRIOBP-6. Key to locations: R1, R2: first and second repeats of TRIOBP-4, Centr.: Central coiled-coil domain of TRIOBP-1. ^5^ Country name, unless a more specific ethnicity was stated in the original paper. ^6^ Or prelingual, but severity not stated. ^7^ Personal communication of additional details by Prof. Shin-ichiro Kitajiri.

**Table 2:** Normal functions and disease states associated with the TRIOBP isoforms.

Function or Phenotype	TRIOBP-1	TRIOBP-4	TRIOBP-5/6 ^1^	Ref.
	Protein Structure			
Principle secondary structure	Helical	Disordered	Disordered	[3,49]
Contains…				
…repeat domains R1 and R1	No	Yes	Yes	[49]
…PH domain	Yes	No	Yes	[3,12]
…coiled-coil domain	Yes	No	Yes	[12,19]
	General function			
Interacts with F-actin	Yes	Yes	Yes	[3,18,50]
Prevents actin depolymerization	Yes	No (?)	?	[12]
Actin bundling activity	No (?)	Yes	Yes (?)	[18,50]
Affects the actin cytoskeleton	Yes	Yes (?)	Yes	[3,45]
Roles in adhesion receptors	Yes	?	?	[13,30]
Implicated in cellular migration	Yes	Yes	Yes	[19,45]
Role in cell cycle progression	Yes	?	?	[21]
	The brain and mental illness			
Expressed in the brain	Yes	No	Yes	[4,5]
Involved in neurite outgrowth	Yes	No	?	[28]
Insoluble (aggregating) in brains of schizophrenia patients	Yes	No (?)	?	[23]
Can aggregate in neurons	Yes	No	Yes	[23]
	Inner ear and deafness			
Expressed in inner ear	Yes	Yes	Yes	[4,5]
Expressed in stereocilia	Yes	Yes ^2^	Yes ^2^	[18,20,50]
Required in stereocilia for				
rootlet formation	No	Yes	No	[50]
Initial bundling of actin	No	Yes	No	[50]
Sculpting and maintenance	No	No	Yes	[18]
Mouse knockout causes deafness?	(Knockout is lethal)	Yes ^3^ (profound)	Yes (progressive)	[18,50]
Mutations in human hearing loss	No (?)	Yes ^3^	Yes	Table 1
	Cancer			
Upregulated in cancer cells?	Many	Specific	Specific	[43,45]
Potential role in metastasis?	Yes	Yes	Yes	[45,46]
	Role in the heart			
Expressed in the heart	Yes	No	No	[4,5]
Function with hERG	Yes	No	No	[15]

^1^ While TRIOBP-5 and 6 likely have at least partially differing roles, no attempt was made to differentiate here due to lack of data. ^2^ Differences in exact role within the stereocilia. ^3^ This mouse knockout and these human mutations would also affect TRIOBP-5/6.
